# ASO Author Reflections: Postoperative Inflammatory Markers as a Surveillance Tool in Colorectal Peritoneal Carcinomatosis

**DOI:** 10.1245/s10434-021-09733-1

**Published:** 2021-03-17

**Authors:** Joey Wee-Shan Tan, Sasinthiran Thiagarajan, Siqin Zhou, Qiu Xuan Tan, Josephine Hendrikson, Wai Har Ng, Gillian Ng, Ying Liu, Jolene Si Min Wong, Grace Hwei Ching Tan, Khee Chee Soo, Melissa Ching Ching Teo, Claramae Shulyn Chia, Chin-Ann Johnny Ong

**Affiliations:** 1 Department of Sarcoma, Peritoneal and Rare Tumours (SPRinT), Division of Surgery and Surgical Oncology, National Cancer Centre Singapore, Singapore, Singapore; 2 Laboratory of Applied Human Genetics, Division of Medical Sciences, National Cancer Centre Singapore, Singapore, Singapore; 3 Duke-NUS Medical School, Singapore, Singapore; 4 Department of Clinical Trials and Epidemiological Sciences, National Cancer Centre Singapore, Singapore, Singapore; 5 SingHealth Duke-NUS Oncology Academic Clinical Program, Duke-NUS Medical School, Singapore, Singapore; 6 Institute of Molecular and Cell Biology, A*STAR Research Entities, Singapore, Singapore

## Past

Despite the volume of literature on the role of inflammatory marker ratios in cancer surveillance,^1–3^ use of these markers in a clinical setting has been limited by considerable heterogeneity seen in publications on selecting cutoffs and the optimal surveillance.^1^^,^^4^ Furthermore, to the best of the authors’ knowledge, there are no reports investigating the course of postoperative inflammatory markers in patients who underwent hyperthermic intraperitoneal chemotherapy (HIPEC) as compared with those who did not.

## Present

In our study,^5^ we addressed an unanswered question on the utility of inflammatory markers in peritoneal carcinomatosis (PC) patients. This is one of the largest studies investigating postoperative inflammatory marker levels at different timepoints in one of the most common peritoneal diseases, colorectal PC. We found that inflammatory markers measured at specific time points during the postoperative follow-up, specifically in CPC, may allow us to identify patients requiring closer monitoring for disease recurrence or poor survival. Furthermore, we found no difference between CPC patients who underwent HIPEC versus those who did not. Low postoperative lymphocyte-monocyte ratio (LMR) (days 4–7), high postoperative neutrophil–lymphocyte ratio (NLR) (days 8–21) and high postoperative platelet–lymphocyte ratio (PLR) (days 22–56) were optimal for prognosticating poor 1-year OS, while high postoperative PLR and NLR (days 57–90) and low postoperative LMR (days 8–21) were associated with poor 1-year RFS. A composite score of these three markers was prognostic for OS in CPC.

## Future

Ideally, our results should be validated in a large cohort in a consortium or a dedicated research centre where there will be comparable multidisciplinary care and chemotherapeutic regimes to control for confounders. The Department of Sarcoma, Peritoneal and Rare Tumours (SPRinT) in the National Cancer Centre Singapore is one such dedicated unit, which aims to deliver cutting-edge research and provide the best possible multidisciplinary care in the Asia–Pacific region. The validation of results could lead to closer monitoring and early intervention for patients at higher risk of mortality and disease recurrence, hence influencing their prognosis. Finally, the authors believe that it is imperative for scientific endeavours to ensure that published data contribute insights to advance the field so that this knowledge can be translated to benefit patients directly in clinical practice.
